# The bottleneck in ENT graduate programs in Brazil

**DOI:** 10.5935/1808-8694.20130002

**Published:** 2015-10-14

**Authors:** Regina Martins, Ubirajara Sennes

**Affiliations:** Senior Associate Professor of Otorhinolaryngology - Medical School of Botucatu (UNESP) and Coordinator of the Graduate Program in Surgery; Associate Professor - Medical School of the University of São Paulo (USP) and Coordinator of the Graduate Program in Otorhinolaryngology - USP

As it is known by everyone, CAPES governs graduate programs in Brazil. It certifies and assesses the programs, classifying them in relation to their excellence at an international level and as far as readership is concerned. The main goal of strictu sensu graduate programs (academic master's degree and PhD) is to train researchers and to create knowledge which can impact science. For that, the first assessment parameter, besides the flow of student titles, is the impact of the publications resulting from the programs' dissertations and theses.

At a first moment, this criterion caused much discomfort among advisors and students, and also many questions: would it be more important to create a publication in a national circulation journal, in Portuguese, which would reach most otorhinolaryngologists in Brazil, or publish in international journals, in English, where but a few Brazilians would read? We believe that both publications are important, for otorhinolaryngologists who work as physicians and who also do research. We have two publications which travel on this road: the Brazilian Journal of Otorhinolaryngology, indexed at Medline and at the JCR (awaiting the impact index), and the International Archives of Otorhinolaryngology - indexed at Scielo.

There are no doubts that this CAPES pressure brought about results, and deserves praise. Brazil improved significantly in the quantity and quality of its publications, today holding the 13th position concerning publications in the world, with a growth curve much higher than that of other countries. Behind this leap in publications are the graduate programs, governed and stimulated by CAPES. Otorhinolaryngology also increased, very much, the quantity and quality of publications, having seen the high and growing number of papers presented in meetings and published in journals, many of them stemming from dissertations and theses, gaining the well-deserved respect and acknowledgement of the world otorhinolaryngology.

On the other hand, otorhinolaryngology has been much harmed by the system utilized to classify the publications. CAPES adopted the so called Qualis system, which classifies them according to their index of impact (number of citations in the journal) within the many fields of knowledge. Otorhinolaryngology belongs to Medicine III - a surgery field, in which Qualis is currently classified into: A1: impact > 3.30, A2 > 2.63, B1 > 1.51, B2 > 0.90, B3 > 0.01, B4: indexed at MedLine, Scielo, LILACS, B5 and C. Although it is a very transparent and honest system, it does not involve the particularities of the surgical field, which is very heterogeneous. Otorhinolaryngology journals have a mean impact of 1.20, which is the lowest in Medicine III (Ophthalmology is 2.73; Anesthesiology is 2.61, Gastric surgery is 2.24; General Surgery is 2.18; Urology is 2.14; Cardiothoracic Surgery is 2.06; Orthopedics is 1.94; Toco-gynechology 1.76 and Plastic surgery 1.42).

Only one ENT journal is Qualis A2 (12 are B1, 14 B2 and 13 B3). Our best and best-known journals, where the most renowned international ENT researchers publish, are B1 and B2. Since they are considered of second line by CAPES, it harms the concept of our graduate programs. However, we should not be compared to researchers from ophthalmology or urology, but rather with our own ENT peers. We have no doubts that we publish in the main journals of our specialty, but one single Qualis to the entire Medicine III is not sensitive to this fact. It is a method which does not fulfill its goal. It compares the otorhinolaryngology researcher with all of those in surgery, but not our peers. The sample is heterogeneous and the conclusions are wrong.

The persistence and idealism pushes us to continue publishing and growing in our specialty, but it is important to state the limitations of the graduate programs' coordinators vis-à-vis the criteria adopted by CAPES.

